# Anthelmintic Activity of Protocatechuic Acid Against Ivermectin-Susceptible and Resistant *Haemonchus contortus* Strains

**DOI:** 10.3390/pathogens15010117

**Published:** 2026-01-21

**Authors:** Jorge Alberto Cortes-Morales, Agustín Olmedo-Juárez, Manasés González-Cortazar, Alejandro Zamilpa, María Eugenia López-Arellano, Humberto Flores-Bustamante, Dante Avilés-Montes, Juan Manuel Rivas-González, César Sotelo-Leyva, David Osvaldo Salinas-Sánchez

**Affiliations:** 1 Laboratorio de Fitoquímica y Productos Naturales del Centro de Investigación en Biodiversidad y Conservación, Universidad Autónoma del Estado de Morelos, Av. Universidad 1001, Col. Chamilpa, Cuernavaca C.P. 62209, Morelos, Mexico; ing_cortesmorales@yahoo.com.mx; 2 Escuela de Estudios Superiores del Jicarero, Universidad Autónoma del Estado de Morelos, Carretera Galeana-Tequesquitengo s/n, Colonia El Jicarero, Jojutla C.P. 62909, Morelos, Mexico; humberto.floresb@uaem.mx (H.F.-B.); manuel.rivas@uaem.mx (J.M.R.-G.); 3 Centro Nacional de Investigación Disciplinaria en Salud Animal e Inocuidad (CENID-SAI, INIFAP), Carretera Federal Cuernavaca-Cuautla No. 8534, Col. Progreso, A.P. 206-CIVAC, Jiutepec C.P. 62574, Morelos, Mexico; olmedo.agustin@inifap.gob.mx (A.O.-J.); mlopez.arellano@gmail.com (M.E.L.-A.); 4 Centro de Investigación Biomédica del Sur, Instituto Mexicano del Seguro Social, Argentina No. 1, Xochitepec C.P. 62790, Morelos, Mexico; azamilpa_2000@yahoo.com.mx; 5 Facultad de Ciencias Biológicas, Universidad Autónoma del Estado de Morelos, Av. Universidad 1001, Col. Chamilpa, Cuernavaca C.P. 62209, Morelos, Mexico; dante.aviles@uaem.mx; 6 Facultad de Ciencias Químico-Biológicas, Universidad Autónoma de Guerrero, Av. Lázaro Cárdenas s/n, Ciudad Universitaria Sur, Chilpancingo C.P. 39000, Guerrero, Mexico; cesarsotelo@uagro.mx

**Keywords:** small ruminant, egg hatch inhibition, larval mortality, anthelmintic resistance, phenolic acids

## Abstract

The frequent and indiscriminate use of all classes of synthetic anthelmintics to deworm small ruminants has decreased their effectiveness in a worldwide problem of anthelmintic resistance. Using active plant metabolites with anthelmintic properties has become a suggested alternative to control parasitic helminths. The present study investigated the ovicidal and larvicidal activity of a fraction (CnF4) containing protocatechuic acid (3,4-dihydroxybenzoic acid) from *Chamaecrista nictitans* (Fabaceae) and a commercial standard of protocatechuic acid against strains of the parasitic nematode *Haemonchus contortus* susceptible (HcIVM-S) and resistant (HcIVM-R) to ivermectin, using egg hatch inhibition (EHI) and L3 larval mortality assays. The CnF4 fraction showed an EHI greater than 90% at 0.8 mg/mL against HcIVM-S and an EHI = 88.39% at 1.6 mg/mL against HcIVM-R. The commercial standard of protocatechuic acid displayed an EHI of 97.49% at 0.25 mg/mL against HcIVM-S and an EHI greater than 98% at 0.5 mg/mL. In the larval mortality assays, protocatechuic acid caused 72.4% larval mortality of HcIVM-S at 8 mg/mL and 53.2% mortality of HcIVM-R at 16 mg/mL. These results indicate that protocatechuic acid was more effective in inhibiting egg hatching and causing larval mortality against HcIVM-S compared to HcIVM-R.

## 1. Introduction

Sheep and goat production is of great economic importance worldwide, mainly for the provision of meat, milk, wool, skin, and their derivatives [1]. Small-scale sheep production is a common activity in rural areas of Mexico, where many people with limited resources depend directly on livestock for their livelihood [2]. Although resistant and resilient animals can be found in production herds, most small ruminants are susceptible to gastrointestinal nematode infections, particularly during the first 3 months of life [3].

Gastrointestinal nematodes (GIN) are the parasites that most frequently affect small ruminants raised in grazing conditions. *Haemonchus contortus* is considered one of the most pathogenic nematodes for sheep and goats [4]. Several reports describe *H. contortus* isolates with resistance to multiple anthelmintic families [5,6]. In this context, producers often rely on commercial anthelmintics such as benzimidazoles, imidazothiazoles, amino-acetonitrile derivatives (i.e., monepantel), and macrocyclic lactones (i.e., ivermectin), or a combination of them. However, the intensive use of these synthetic drugs favours the development of anthelmintic resistance [7,8]. In fact, over the last two decades, strains of nematodes resistant to multiple families of anthelmintics have emerged.

The macrocyclic lactones (i.e., ivermectin, moxidectin) anthelmintic resistance among GIN populations has been associated with several molecular mechanisms, including genomic modifications in the binding sites on the chloride ion channels, enzymatic detoxification pathways, and xenobiotic efflux mediated by P-glycoproteins [9,10].

The emergence of multiple anthelmintic resistance has encouraged several research groups to explore alternative control strategies such as the selection of genetically resistant animals, vaccines, pasture rotation, biological control agents, and forage plants with anthelmintic activity [11,12,13]. Forage plants contain specialised metabolites such as tannins, flavonoids, hydroxycinnamic acids, and terpenes capable of interrupting the biological cycle of GIN at some stage of their life cycle. Natural products from plants or fungi offer promising opportunities for integrating GIN control strategies. Some studies have even evaluated combinations of commercial anthelmintics like albendazole with terpenes (i.e., carvacrol) or ivermectin with quercetin, demonstrating possible synergistic effects against *H. contortus* isolates with multiple anthelmintic resistance [14,15].

Recently, our research group evaluated the ovicidal activity of *Chamaecrista nictitans* (Fabaceae) extract and fractions against the parasitic nematode *Haemonchus contortus* by using two bioactive fractions of ferulic acid and coumaric acid. Two strains of *H. contortus* were tested, one susceptible and one resistant to ivermectin. The resistant strain required higher concentrations of these compounds (effective concentrations: 57.5 vs. 25.9 ug/mL for ferulic acid and 51.1 vs. 24.4 µg/mL for *p*-coumaric acid, respectively) to achieve the same percentage of egg-hatch inhibition obtained against the susceptible strain [16].

Protocatechuic acid is a phenolic acid belonging to the hydroxybenzoic acid group and has been identified in several family plants (Fabaceae and Anacardiaceae) with reported anthelmintic activity [17,18]. In our research group, this phenolic acid was identified as a major compound in a bioactive fraction (CnF4) obtained from *C. nictitans* aerial parts and displayed important anthelmintic activity on *H. contortus* (unpublished data).

Protocatechuic acid has exhibited several medicinal properties like anticancer, antioxidant and antibacterial activities and several experiments have shown that it is not cytotoxic at moderate concentrations [19,20]. This compound had demonstrated nematocidal activity against the root parasitic nematode *Meloidogyne incognita* [21]. In another study by de Jesús-Martínez et al. [18], they identified this phenolic acid in a bioactive subfraction from *Cyrtocarpa procera* bark and an important ovicidal and larvicidal on *H. contortus* was observed.

Therefore, the objective of the present study was to evaluate the ovicidal and larvicidal activity of the CnF4 fraction and a commercial standard of protocatechuic acid against strains of *H. contortus* susceptible and resistant to ivermectin.

## 2. Materials and Methods

### 2.1. Plant Material

*C. nictitans* plants were collected from Quilamula, Tlaquiltenango, Morelos, México (N 18°30′41″, W 99°00′40″, 1143 m above sea level) in October 2023. A specimen with voucher number 39819 was deposited in the Herbarium of the Biodiversity and Conservation Research Centre. Aerial parts (12 kg) were dried in the shade until constant weight (1.8 kg) was attained and ground with an industrial mill. CnF4 fraction was obtained from an ethyl acetate (EtOAc) extract of aerial parts of *C. nictitans* by bioguided fractionation described previously [16].

### 2.2. High-Performance Liquid Chromatography Analyses

The protocatechuic acid identification in CnF4 was carried out using a high-performance liquid chromatography system (Delta Prep 4000, Waters, Milford, MA, USA) equipped with a Waters 2695 separation module, photodiode array detector (Waters 996), and Pro Empower^TM^ 3 software (Waters 2010) according to Gutiérrez-Román et al. [22]. Fraction samples of 10 µL (2 mg/mL) were separated in a reverse phase Supelcosil LC-F column (250 mm × 4 mm and 5 µm particle size) (Sigma-Aldrich, Bellefonte, PA, USA) connected to a guard column. The elution was carried out using a mobile phase consisting of 0.5% trifluoroacetic acid aqueous solution (solvent A) and acetonitrile (solvent B), maintaining a flow rate of 0.9 mL/min and wavelength range detection of 190–600 nm. The gradient system was as follows: 0–1 min, 0% solvent B; 2–3 min, 5% solvent B, 4–20 min, 30% solvent B; 21–23 min, 50% solvent B; 24–25 min, 80% solvent B; 26–27 100% solvent B; 28–30 min, 0% solvent B. Absorbance was measured at 280 nm to identify hydroxybenzoic acid derivatives. The identification of this compound was performed by comparison of retention time and UV absorption spectrum with the commercial standard protocatechuic acid (code sc-205818, Santa Cruz Biotechnology Inc., Dallas, TX, USA) that was used for the bioassays in the present research. The quantification of protocatechuic acid in the CnF4 fraction was performed using a calibration curve of this compound as a standard at increasing concentrations of 12.5, 25, 50, 100, and 200 mg/mL, dissolved in HPLC-grade methanol (Table S1, Figure S1). Linearity was determined by linear regression of the calibration curves, based on the relationship between the area under the curve (AUC) of the CnF4 fraction peak and the same protocatechuic acid signal. Sensitivity was defined as the lowest detectable concentration and lowest limit of detection, based on the signal-to-noise ratio (S/N) of the CnF4 peaks (Figure S2), obtained using the lower limit of quantification (LLOQ, 48.7 µg/mL) and lower limit of detection (LLOD, 16.06 µg/mL). The LLOQ must meet the accuracy and precision criteria, evaluated using a relative standard deviation (RSD) of 20%.

### 2.3. Nematode Material

Eggs and larvae (L3) of *H. contortus* were recovered from faeces collected directly from the rectum of two donor sheep (22 and 19 kg body weight) previously infected with 6600 and 7700 *H. contortus* infective larvae (L3) susceptible (HcIVM-S) and resistant (HcIVM-R), respectively, to ivermectin. The sheep were housed in individual cages, fed with commercial feed, hay, and alfalfa, and given access to water ad libitum. HcIVM-S (INIFAP-HcIVMs-SAI, Morelos, Mexico) was originally obtained from a herd of naturally infected lambs at Las Margaritas Ranch in Hueytamalco, Puebla State, Mexico, in 1990 [23]. The molecular characterisation of INIFAP-HcIVMs-SAI was reported by Reyes-Guerrero et al. [23,24]. HcIVM-R (INIFAP-HcIVMr-SAI, Morelos, Mexico) was obtained from a naturally infected grazing sheep with resistance problems to ivermectin in the Salto del Agua District, Chiapas, Mexico [25]. The resistance of HcIVM-R was confirmed by a faecal egg count reduction test, in vitro assays, and the polymerase chain reaction (PCR) technique [24,26,27]. Both strains are preserved under cryopreservation conditions at the National Centre for Disciplinary Research in Animal Health and Innocuity of INIFAP-Agricultura. Nucleotide sequence data for these strains are available in the GenBankTM database (BioProject number: PRJNA877658). For the evaluation of the phenolic acid, both *H. contortus* strains were maintained at our National Center in donor animals (four lambs per strain into the year). For ivermectin-resistant strain, donor lambs are periodically treated with sub therapeutic doses of ivermectin to maintain selection pressure and preserve the genetic integrity of the resistant phenotype.

Egg recovery was carried out according to the technique described by Coles et al. [28] with minor modifications. Thirty-five grams of faeces were macerated and homogenised with tap water. The faecal macerate was distributed among 12 50 mL Falcon tubes (35 mL each) containing 15 mL of saturated saline solution (42%). The tubes were shaken to suspend the eggs and centrifuged at 3500 rpm for 5 min. The supernatant was washed with clean water and sieved through 72 and 32 µm meshes to collect the eggs in a 15 mL Falcon tube. Dilutions were made to obtain a stock egg solution of 100 ± 15 eggs/50 μL to be used in the ovicidal bioassay [18].

To obtain L3, faeces were collected in a basin for 24 h from the egg-donor sheep. Cultures were prepared by macerating and mixing the faeces with tap water, using polystyrene particles in a basin to obtain adequate oxygenation and optimal hatching of larvae from the eggs. The faecal cultures were covered with aluminium foil and incubated for 7 days at 25–31 °C. Infective L3 were extracted from the faecal cultures by using the Baermann funnel technique and cleaned by density gradient in distilled water with a paper filter and centrifugation. Finally, the L3 were subjected to a sheathing process with 0.187% sodium hypochlorite and washed with distilled water. Dilutions were made to obtain a stock larval solution of 100 ± 15 L3/50 μL to be used in the larval mortality bioassay [29].

### 2.4. Egg Hatching Inhibition Test

The egg hatch inhibition (EHI) test was performed to evaluate the CnF4 and commercial standard of protocatechuic acid (sc-205818) against HcIVM-S and HcIVM-R. The test was conducted in triplicate in 96-well micro-titration plates, with four repetitions for each concentration evaluated (*n* = 12 for each strain) [30]. The treatments were designed as follows. The CnF4 was tested at 0.2, 0.4, 0.8, and 1.6 mg/mL against HcIVM-S and HcIVM-R. The protocatechuic acid was tested at 0.031, 0.062, 0.125, and 0.25 mg/mL against HcIVM-S and at 0.125, 0.25, 0.5, and 1 mg/mL against HcIVM-R. Distilled water and 3% methanol (to dissolve the CnF4 fraction and commercial standard) were used as negative controls, and thiabendazole at 0.1 mg/mL (dissolved in dimethyl sulfoxide) as a positive control. For biological evaluation, 50 µL of the aqueous suspension containing 100 ± 15 eggs was deposited in each well. Then, 50 µL of CnF4, protocatechuic acid, or a control was deposited in separate wells. The microtiter plates were incubated at room temperature (25 ± 3 °C) for 48 h. The number of eggs and larvae in each well was counted under an optical microscope at 10×. The percentage of egg hatch inhibition (%EHI) for each treatment was determined according to the following formula:

$$\%EHI=\frac{number\;of\;eggs}{number\;of\;eggs+number\;of\;larvae}\times 100$$

### 2.5. Larval Mortality Test

The larval mortality bioassay of CnF4 and the commercial standard of protocatechuic acid (sc-205818) against HcIVM-S and HcIVM-R L3 was carried out in 96-well microtiter plates in triplicate, with four repetitions per concentration (*n* = 12 for each strain) [24]. The CnF4 was evaluated at 6.25, 12.5, 25, and 50 mg/mL against HcIVM-S and HcIVM-R, while protocatechuic acid was evaluated at 5, 6, 7, and 8 mg/mL against HcIVM-S and 2, 4, 8, and 16 mg/mL against HcIVM-R. Distilled water and polyvinylpyrrolidone (PVP, to dissolve the CnF4 fraction and commercial standard) at 6 mg/mL were used as negative controls, while ivermectin (5 mg/mL) was used as a positive control. For biological evaluation, 50 µL of the aqueous suspension containing 100 ± 15 infective L3 were added to each well, and then aliquots of 50 μL of CnF4, protocatechuic acid, and the controls were deposited in separate wells. The plates were incubated for 72 h at 25 ± 3 °C. A count of dead and live L3 was performed under an optical microscope to determine the percentage of L3 mortality (%LM) using the following formula:

$$\%LM=\frac{number\;of\;dead\;L3}{number\;of\;dead\;L3+number\;of\;live\;L3}\times 100$$

### 2.6. Statistical Analysis

The EHI and larval mortality percentages were normalised using an arcsine square root transformation and analysed using a completely randomised factorial design through analysis of variance and general linear models in SAS 9.0 [31]. Analysed factors were: (1) Compound (CnF4 and protocatechuic acid commercial standard), (2) Strain (HcIVM-S and HcIVM-R) and Concentration (0.031–16 mg/mL). Tukey’s test (alpha < 0.05) was performed to determine significant differences between treatment means. Treatments with a concentration-dependent effect were subjected to a regression analysis to determine the effective concentrations for 50% and 90% mortality (EC_50_ and EC_90_, respectively), using the Proc Probit analysis in SAS 9.0.

## 3. Results

### 3.1. Chemical Identification of the C. nictitans Bioactive Fraction

**Figure 1:**
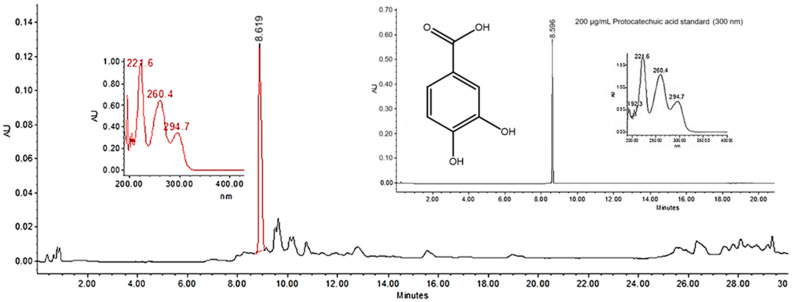
High-performance liquid chromatogram of the CnF4 bioactive fraction and a commercial standard of protocatechuic acid (insert) recorded at 300 nm. The red color line refers to the protocatechuic acid peak in the CnF4 fraction.

The CnF4 fraction yielded 1.35 g from the complete extract. The chromatogram of the CnF4 fraction displayed a single main peak (Figure 1). The presence of protocatechuic acid at Rt = 8.619 min (λ_nm_ 221, 260, 294) was determined by analysing its UV absorption spectra and comparison with the database and the protocatechuic acid commercial standard (sc-205818) as reference (Figure 1). According to the area under the peak curve in the CnF4 fraction and the calibration curve of the protocatechuic acid commercial standard, the quantification of this compound was 0.081 mg per mg of CnF4.

### 3.2. Egg Hatching Inhibition Test

Table 1 shows the EHI percentages of HcIVM-S and HcIVM-R strains after 48 h of exposure to the CnF4 fraction, protocatechuic acid, and control treatments. The CnF4 showed an EHI greater than 90% at 0.8 mg/mL against HcIVM-S and an EHI of 88.39% at 1.6 mg/mL against HcIVM-R. The protocatechuic acid displayed an EHI of 97.49% at 0.25 mg/mL against HcIVM-S and an EHI greater than 98% at 0.5 and 1 mg/mL. The EHI by the negative controls (distilled water and methanol 3%) was less than 2.34% and greater than 99% by the thiabendazole treatment against both strains.

Table 2 shows the EC_50_ and EC_90_ of CnF4 and protocatechuic acid inhibiting HcIVM-S and HcIVM-R egg hatch. The CnF4 fraction produced an EC_50_ = 0.368 mg/mL against HcIVM-S, which was 1.3-fold lower than that obtained against HcIVM-R (0.489 mg/mL). The CnF4 produced an EC_90_ = 0.697 mg/mL against HcIVM-S, which was 1.9-fold lower than that obtained with HcIVM-R (1.348 mg/mL). On the other hand, the protocatechuic acid EC_50_ was 0.054 mg/mL, and the EC_90_ was 0.118 mg/mL against HcIVM-S, which was 3.8 and 3.2-fold lower than the EC_50_ (0.207 mg/mL) and EC_90_ (0.380 mg/mL), respectively, against HcIVM-R.

### 3.3. L3 Mortality Test

Table 3 shows the L3 mortality percentages of HcIVM-S and HcIVM-R after 72 h of exposure to CnF4, protocatechuic acid, and control treatments. The CnF4 at 50 mg/mL caused 35% mortality of HcIVM-S. Mortality of HcIVM-R exposed to 50 mg/mL of CnF4 was less than 12%. The protocatechuic acid at 8 mg/mL produced 72.4% mortality of HcIVM-S and 53.2% mortality at 16 mg/mL against HcIVM-R. The negative controls (distilled water and PVP in methanol 3%) caused less than 3.4% mortality, and ivermectin produced mortality greater than 99% against both strains.

Since the CnF4 showed larvicidal activity of less than 50% against both strains, it was impossible to calculate the 50% (LC_50_) and 90% lethal concentrations. The LC_50_ for protocatechuic acid was 6.16 mg/mL against HcIVM-S. Due to the low larvicidal activity against HcIVM-R, it was also not possible to calculate the 90% lethal concentrations.

## 4. Discussion

The search for natural products with anthelmintic potential has become important in the livestock sector for controlling parasites that affect domestic animals. Plants in the family Fabaceae have been shown to contain chemical constituents capable of interrupting the biological cycle of gastrointestinal nematodes, including *H. contortus* [32,33,34]. The results of our research indicate that the protocatechuic acid commercial standard identified in CnF4 fraction from the Cn-AcOEt extract of *C. nictitans* exerts ovicidal and larvicidal activity on *H. contortus* strains susceptible and resistant to ivermectin.

The CnF4 fraction at 0.8 mg/mL showed a 1.2-fold greater EHI against HcIVM-S (93.74%) compared to HcIVM-R (77.42%). Similarly, the commercial standard of protocatechuic acid at 0.25 mg/mL showed a 1.4-fold greater EHI against HcIVM-S (97.49%) compared to HcIVM-R (69.86%). Analysing the EC_90_ values, it is notable that the commercial standard of protocatechuic acid was 5.8 and 3.5-fold more effective against HcIVM-S and HcIVM-R, respectively, than the Cn4F fraction. Contrary to our findings, Al-Rofaai et al. [35] did not observe significant differences in EHI when evaluating methanolic extracts of leaves of *Azadirachta indica* (Meliaceae) and *Manihot esculenta* (Euphorbiaceae) against strains of *Teladorsagia circumcincta* susceptible and resistant to macrocyclic lactones. Al-Rofaai et al. [36] evaluated a methanolic extract of *M. esculenta* against the eggs and larvae of strains of *Trichostrongylus colubriformis* susceptible and resistant to macrocyclic lactones. They observed no significant differences between the susceptible and resistant strains in either EHI or larval mortality bioassays. The quantification of protocatechuic acid in the fraction indicates that each 1 mg of the CnF4 fraction contains 0.081 mg of protocatechuic acid.

It is important to note that, despite being a major component, protocatechuic acid was not purified from the CnF4 fraction. The quantification of protocatechuic acid in the fraction indicates that each 1 mg of the CnF4 fraction contains 0.081 mg of protocatechuic acid. In other words, each concentration of the CnF4 fraction evaluated in this study contains only 8% protocatechuic acid, meaning that the CnF4 fraction must contain traces of other chemical compounds that contribute additively or synergistically to the biological activity.

In our study, the CnF4 fraction showed low L3 mortality, 35% against HcIVM-S and 11% against HcIVM-R at 50 mg/mL; this result may be because the fraction did not have an adequate concentration of protocatechuic acid (4.05 mg) to cause high L3 mortality. However, the commercial standard of protocatechuic acid at 8 mg/mL showed 1.8 times higher mortality against HcIVM-S compared to HcIVM-R. These results differ from those reported by de Araújo-Filho et al. [37], who evaluated an essential oil of *Eucalyptus citriodora* (Myrtaceae) with high citronellol content against eggs and larvae of *H. contortus* strains susceptible and resistant to macrocyclic lactones. They demonstrated that both the oil and citronellol caused 100% larval mortality at 2 mg/mL against the susceptible and resistant strains. On the other hand, Frota et al. [38] evaluated the compounds geraniol, citronellol, anacardic acid, and cinnamaldehyde against eggs and larvae of an *H. contortus* strain susceptible and resistant to macrocyclic lactones. They reported that all compounds effectively inhibited egg hatch rate and larval development against both strains, with no significant difference between strains; anacardic acid and cinnamaldehyde showed the highest activity.

Protocatechuic acid is a water-soluble phenolic acid that has shown several pharmacological and antimicrobial activities, such as antifungal, antibacterial, and anthelmintic [21,39,40]. Our study is the first to report the ovicidal and larvicidal activity of protocatechuic acid against *H. contortus* strains susceptible and resistant to ivermectin.

Pavičić et al. [41] demonstrated the ovicidal and larvicidal activity of methanolic extracts of eight European ferns against *H. contortus*. The analysis of polyphenols from three of the plant species revealed that the compounds present in the extracts were mostly derivatives of flavonoids, followed by derivatives of hydroxycinnamic acids and derivatives of hydroxybenzoic acids, including protocatechuic acid. Nguyen et al. [21] isolated protocatechuic acid from the bark of *Terminalia nigrovenulosa* (Combretaceae) and evaluated its nematicidal activity against the plant parasitic nematode *Meloidogyne incognita*; they reported that protocatechuic acid at 1 mg/mL produced 85% EHI and 94% mortality. Auniq et al. [42] reported high nematicidal activity of an ethanol extract from the leaves of *Vitex peduncularis* (Lamiaceae) against the annelid *Tubifex tubifex*, while in silico molecular docking analysis revealed that protocatechuic acid displayed moderate affinity for the tubulin-colchicine enzyme complex, which predicts the anthelmintic activity of this compound.

Within our research group, we have also identified protocatechuic acid present in extracts and fractions from other plants. For example, De Jesús-Martínez et al. [18] demonstrated the ovicidal and larvicidal activity of a hydroalcoholic extract and fractions from the fruits of *Cyrtocarpa procera* (Anacardiaceae) against *H. contortus*; phytochemical analysis revealed the presence of gallic acid, ellagic acid, and protocatechuic acid in the active fractions. Becerril-Gil et al. [43] demonstrated the ovicidal activity of an EtOAc extract and its fractions from the aerial parts of *Arceuthobium vaginatum* (Santalaceae) against *H. contortus*. Phytochemical analysis revealed the presence of glycosylated flavonoid derivatives, hydroxycinnamic acid derivatives, coumarins, and hydroxybenzoic acids, including protocatechuic acid. Currently, there are no studies on the resistance mechanism of *H. contortus* to plant secondary metabolites. The reduced anthelmintic activity of protocatechuic acid against the resistant strain of *H. contortus* suggests that some of the ivermectin resistance mechanisms, such as metabolic detoxification via drug-metabolising enzymes, alterations in glutamate-gated chloride channels in nerve and muscle cells, or alterations in membrane-bound P-glycoproteins (P-gp), are being activated. However, studies have shown that combining phenolic compounds with drugs blocks P-gp function, improving the drug’s biological activity [44,45]. Borges et al. [46] evaluated the anthelmintic activity of the flavonoid quercetin individually, and in combination with ivermectin; the anthelmintic activity of ivermectin improved when combined with quercetin. The authors hypothesised that quercetin acted as a blocker of the P-gp function, increasing the concentration of ivermectin within the cell and its biological activity.

## 5. Conclusions

This research demonstrated that the commercial standard of protocatechuic acid has ovicidal and larvicidal activity against *H. contortus* on both the susceptible and ivermectin-resistant strains; however, the ivermectin-resistant strain of *H. contortus* requires a higher concentration of protocatechuic acid to produce the same lethal effect as on the susceptible strain, which indicates that *H. contortus* exhibits a anthelmintic resistance mechanism to phenolic compounds, such as protocatechuic acid-like. Biochemical and molecular studies are needed to determine whether the resistance mechanism of *H. contortus* to protocatechuic acid is similar to the ivermectin resistance mechanism.

## Figures and Tables

**Table 1:** Egg hatch inhibition percentages induced by the CnF4 fraction from *Chamaecrista nictitans* and protocatechuic acid commercial standard against two *Haemonchus contortus* strains (resistant and susceptible to ivermectin) after an incubation period of 48 h.

Treatments	Concentration (mg/mL)	EHI (% ± s.d)	
		HcIVM-S	HcIVM-R
Distilled water		2.34 ± 1.73 ^e^	1.43 ± 1.58 ^f^
Methanol 3%		3.13 ± 1.92 ^e^	1.35 ± 0.97 ^f^
Thiabendazole	0.1	99.67 ± 0.51 ^a^	99.45 ± 0.55 ^a^
CnF4	1.6	98.29 ± 0.81 ^a,b^	88.39 ± 3.26 ^b^
	0.8	93.74 ± 2.78 ^b^	77.42 ± 2.72 ^c^
	0.4	67.72 ± 5.80 ^c^	52.03 ± 2.07 ^d^
	0.2	5.40 ± 1.85 ^e^	4.91 ± 1.88 ^e,f^
Protocatechuic acid commercial standard	1	100 ^a^	98.93 ± 0.35 ^a^
	0.5	100 ^a^	99.84 ± 0.19 ^a^
	0.25	97.49 ± 1.2 ^a,b^	69.86 ± 11.12 ^c^
	0.125	51.17 ± 2.65 ^d^	13.07 ± 11.16 ^e^
	0.062	47.45 ± 2.31 ^d^	2.63 ± 0.69 ^ef^
	0.031	46.19 ± 1.52 ^d^	nt
*p* value (Compound * strain * concentration)		<0.0001	
Variation coefficient		9.45	
R^2^		0.98	

^a–f^ Means with different letters in the same column represent statistical differences, *p* < 0.05. s.d = standard deviation, *n* = 12. nt = not tested. * = interaction among factors.

**Table 2:** Effective concentrations to inhibit Haemonchus contortus egg hatching by 50% and 90% (EC_50_ and EC_90_, respectively) of resistant and susceptible strains to ivermectin after a 48-h incubation period with the CnF4 fraction from Chamaecrista nictitans and protocatechuic acid commercial standard.

Treatments	EC_50_ (mg/mL)				EC_90_ (mg/mL)			
	HcIVM-S		HcIVM-R		HcIVM-S		HcIVM-R	
CnF4	0.368 95% CI limits		0.489 95% CI limits		0.697 95% CI limits		1.348 95% CI limits	
	Lower 0.35	Upper 0.38	Lower 0.46	Upper 0.51	Lower 0.66	Upper 0.74	Lower 1.25	Upper 1.47
Protocatechuic acid commercial standard	0.054 95% CI limits		0.207 95% CI limits		0.118 95% CI limits		0.380 95% CI limits	
	Lower 0.051	Upper 0.056	Lower 0.20	Upper 0.21	Lower 0.11	Upper 0.13	Lower 0.36	Upper 0.39

CI = Confidence Interval.

**Table 3:** Larval mortality percentages induced by the CnF4 fraction from *Chamaecrista nictitans* and protocatechuic acid commercial standard against two *Haemonchus contortus* strains (resistant and susceptible to ivermectin) after an incubation period of 72 h.

Treatments	Concentration (mg/mL)	Mortality (% ± s.d)	
		HcIVM-S	HcIVM-R
Distilled water		3.43 ± 2.15 ^g^	1.12 ± 0.81 ^d^
PVP in Methanol 3%		2.81 ± 1.21 ^g^	1.53 ± 1.65 ^d^
Ivermectin	5	100 a	96.61 ± 2.58 ^a^
CnF4	50	35.07 ± 5.07 ^cd^	11.85 ± 4.56 ^d^
	25	25.34 ± 5.16 ^de^	8.50 ± 3.51 ^d^
	12.5	10.96 ± 2.84 ^fg^	4.28 ± 2.49 ^d^
	6.25	3.97 ± 1.09 ^g^	3.27 ± 2.42 ^d^
Protocatechuic acid standard commercial	16	nt	53.21 ± 14.68 ^b^
	8	72.48 ± 4.64 ^b^	39.42 ± 17.42 ^bc^
	7	70.48 ± 11.57 ^b^	nt
	6	44.13 ± 15.27 ^c^	nt
	5	23.97 ± 6.51 ^def^	nt
	4	15.05 ± 2.46 ^efg^	32.71 ± 15.22 ^c^
	2	nt	20.65 ± 12.04 ^cd^
*p* value (Compound * strain * concentration)		<0.0001	
Variation coefficient		28.58	
R^2^		0.94	

^a–g^ Means with different letters in the same column represent statistical differences *p* < 0.05. s.d = standard deviation. *n* = 12. nt = not tested. * = interaction among factors

## Data Availability

The original contributions presented in this study are included in the article/Supplementary Material. Further inquiries can be directed to the corresponding authors.

## Supplementary Materials

The following supporting information can be downloaded at: https://www.mdpi.com/article/10.3390/pathogens15010117/s1, Figure S1: Linear regression of the calibration curve of the protocatechuic acid commercial standard; Figure S2: Calculation of the limit of detection (LOD) and limit of quantification LOQ from Regression Data; Table S1: Calibration curve of the protocatechuic acid commercial standard.
