# Supplementary material for: Anthelmintic Activity of Protocatechuic Acid Against Ivermectin-Susceptible and Resistant Haemonchus contortus Strains
Source: Pathogens. 2026 Jan 21;15(1):117. doi: 10.3390/pathogens15010117 (PMC12845097; doi:10.3390/pathogens15010117)
Supplement: Supplementary file 1 [file pathogens-15-00117-s001.zip › pathogens-4028815-supplementary.pdf]

Supplementary data of the manuscript

"Anthelmintic activity of protocatechuic acid against ivermectin susceptible and resistant *Haemonchus contortus* strains"

Table S1 Calibration curve of the protocatechuic acid commercial standard

Concentration (mg/mL)	Retention Time (TR)	Area 1	Area 2	Area 3	Average area
12.5	8.599	128261	128261	128261	128261
25	9.25	768377	767968	769117	768487.3333
50	9.24	1539199	1521176	1510430	1523601.667
100	9.28	3209653	3156332	3221963	3195982.667
200	9.24	6727183	6680140	6626845	6678056

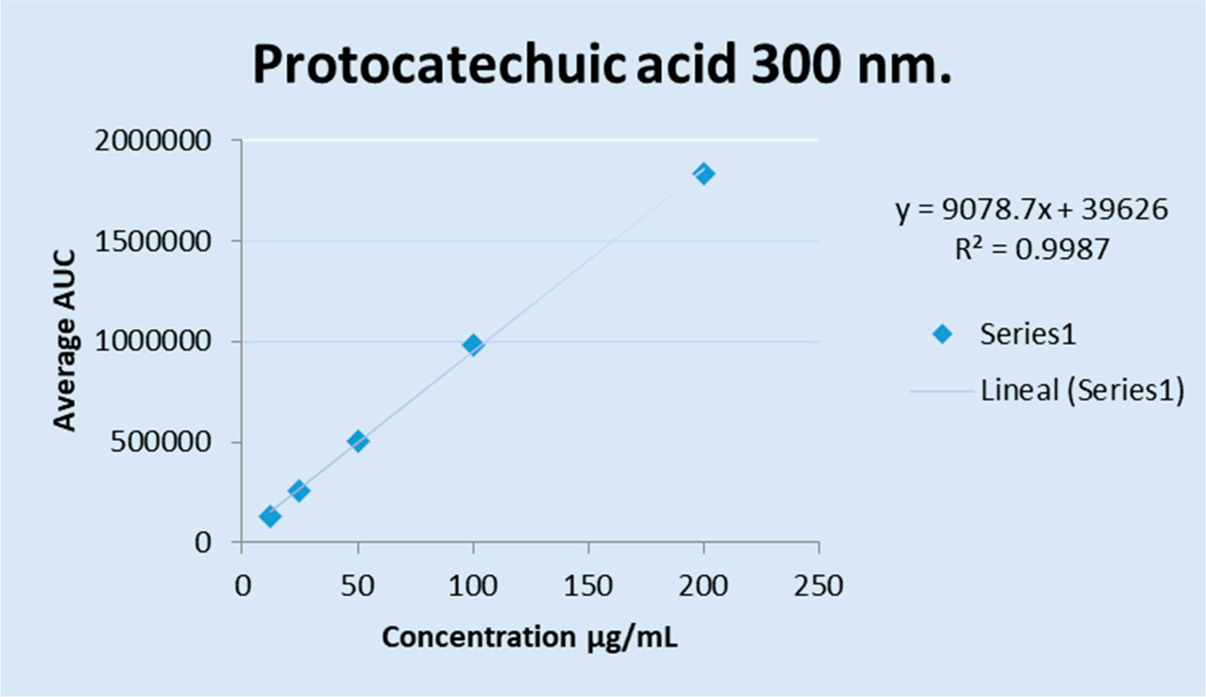

Figure S1 Linear regression of the calibration curve of the protocatechuic acid commercial standard

**SE of intercept**	19759.58118
**SD of intercept**	44183.76674
**LOD**	**16.06027628**
**LOQ**	**48.66750387**

Y= ax + b

Slope: **a** = **9078.7**

Intercept **b**

The number of test **N** = 5

LOD = 3.3 * (SD of intercept/slope)

LOQ = 10 * (SD of intercept/slope)

Summary output

Regresion statistics	
Multiple R	0.99933387
R^2	0.99866818
Adjusted R^2	0.99822424
Standard error	29186.8357
Observations	5

ANOVA

	df	*SS*	*MS*	*F*	*Significance F*
Regresion	1	1.91634E+12	1.9163E+12	2249.561119	2.06362E-05
Residual	3	2555614136	851871379		
Total	4	1.91889E+12			

	Coefficients	*Standard Error*	*t Stat*	*P-value*	*Lower 95%*	*Upper 95%*	*Lower 95.0%*	*Upper 95.0%*
Intercept	39625.9722	19759.58118	2.00540547	0.138598375	-23257.83391	102509.778	-23257.834	102509.778
X Variable 1	9078.71606	191.4148092	47.4295385	2.06362E-05	8469.548705	9687.88341	8469.5487	9687.88341

Figure S2 Calculation of the limit of detection (LOD) and limit of quantification LOQ from Regression Data
